# Development of visual Neuroprostheses: trends and challenges

**DOI:** 10.1186/s42234-018-0013-8

**Published:** 2018-08-13

**Authors:** Eduardo Fernandez

**Affiliations:** 1 Institute of Bioengineering, University Miguel Hernández and CIBER-BBN, Avda de la Universidad, s/n, 03202 Alicante, Elche Spain; 2 John A. Moran Eye Center, University of Utah, Salt Lake City, USA

**Keywords:** Artificial vision, Restoration of sight, Blindness, Phosphene, Brain plasticity

## Abstract

Visual prostheses are implantable medical devices that are able to provide some degree of vision to individuals who are blind. This research field is a challenging subject in both ophthalmology and basic science that has progressed to a point where there are already several commercially available devices. However, at present, these devices are only able to restore a very limited vision, with relatively low spatial resolution. Furthermore, there are still many other open scientific and technical challenges that need to be solved to achieve the therapeutic benefits envisioned by these new technologies. This paper provides a brief overview of significant developments in this field and introduces some of the technical and biological challenges that still need to be overcome to optimize their therapeutic success, including long-term viability and biocompatibility of stimulating electrodes, the selection of appropriate patients for each artificial vision approach, a better understanding of brain plasticity and the development of rehabilitative strategies specifically tailored for each patient.

## Introduction

Few disabilities affect human life and personal fates more than the loss of the ability to see. This problem affects to more than 40 million people worldwide and is associated with loss of personal independence, inducing large personal and societal costs. Although some of these patients can be effectively treated with surgery or medication, and some recent developments in gene therapies and stem cell therapies are showing a great promise (Higuchi et al., 2017; Llonch et al., 2018; Artero Castro et al., 2018), unfortunately there are no effective treatments for many persons that are visually handicapped as a result of severe degeneration or damage to the retina, the optic nerve, or the brain. In such cases, a visual prosthesis may be the only option. Similar assistive devices have already allowed thousands of deaf patients to hear sounds and acquire language abilities, and the same hope exists in the field of visual rehabilitation (Introduction to Visual Prostheses [Internet], 2018; Mills et al., 2017; Brandli et al., 2016; Weiland et al., 2016).

**Fig. 1:**
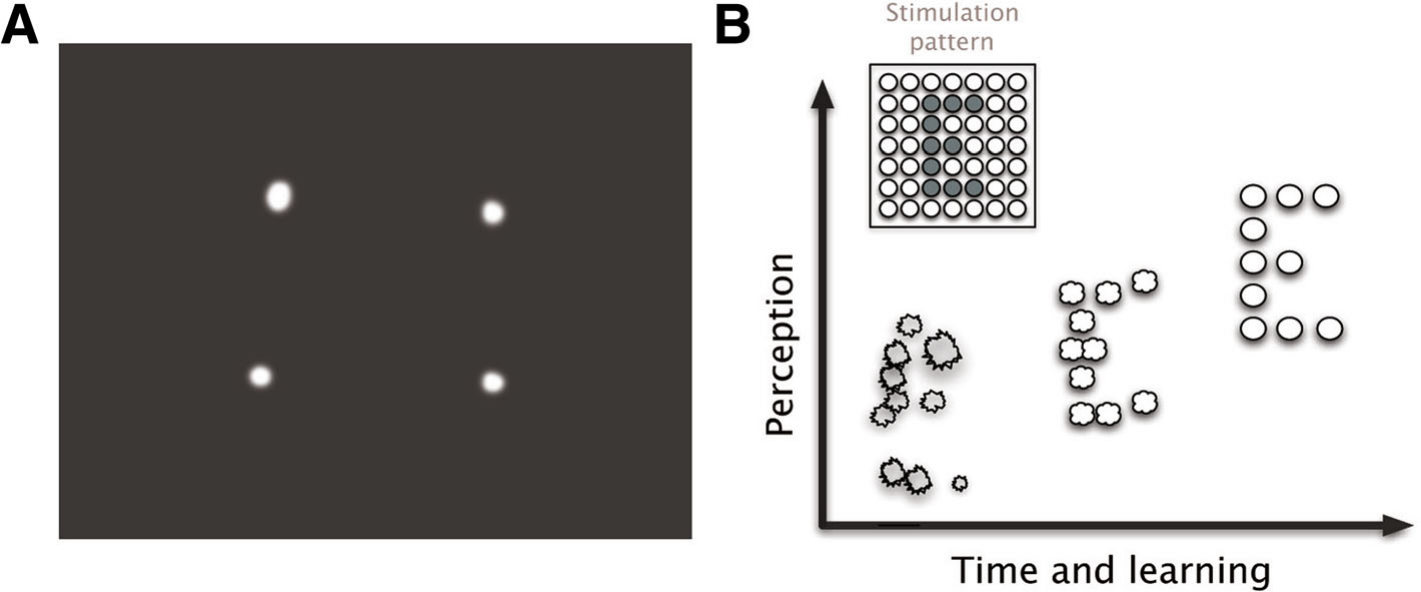
Examples of patterned phosphenes. **a**: Possible perception generated by stimulating simultaneously 4 electrodes arranged as a square. **b**: The neural plasticity of the visual system can contribute to ever-improving correlation between the physical world and evoked phosphenes. Immediately after implantation the evoked phosphenes are likely to induce a poor perception of an object (the letter “E” in this example). However, appropriate learning and rehabilitation strategies will contribute to provide concordant perceptions

The possibility to restore sight in blind individuals has a long history in biomedical engineering, but the modern era of artificial vision research started with a German neurosurgeon, Otfrid Foerster, who was the first to expose the human occipital pole under local anesthesia and to electrically stimulate it (Foerster, 1929). In 1929 he noted that electrocortical stimulation induced the perception of punctate sensations of light, called technically phosphenes, which were usually described as ‘stars in the sky’, ‘clouds’ and ‘pinwheels’. These findings together with the earlier studies of Wilder Penfield and co-workers during the course of neurosurgical interventions for the treatment of epilepsy (Penfield & Jaspers, 1974; Penfield & Rasmussen, 1950), established the physiological basis for present efforts to develop a visual prosthesis to substitute, and ultimately restore sight. Subsequent experiments by the group of Giles Brindley in England, William Dobelle at the University of Utah, Pollen and others, that have been recently reviewed by Lewis et al. (Lewis et al., 2015), opened a new period in the field and showed that stimulation of multiple electrodes simultaneously was able to allow blind volunteers to recognize simple patterns, including letters and Braille characters (Fig. 1). Thus, even if a crude representation can be delivered to the visual cortex, the blind subject can eventually be able to use this information to create an image that comports with his or her sense of the surrounding physical world.

The results of these studies supported the premise that patterned electrical stimulation of the visual cortex can evoke patterned percepts. However, these initial efforts did not culminate in the restoration of a useful visual sense. Problems with these early works have been associated mainly with the large surface electrodes that were used to evoke phosphenes (Normann et al., 1996). Thus, relatively high electrical currents were required to evoke phosphenes and when multiple electrodes were stimulated, these large currents could interact in a non-linear fashion, evoking phosphenes with unpredictable spatial properties. Furthermore, there were also occasional elicitation of pain due to meningeal or scalp stimulation. All of this, together with the risk of inducing epileptic seizures has led a number of investigators to develop other types of visual prostheses designed to be implanted directly into the retina, the optic nerve or other parts of the visual pathways (Introduction to Visual Prostheses [Internet], 2018; Brandli et al., 2016).

This technology has developed tremendously over the last years and today artificial vision is an exciting subject in both ophthalmology and basic science that has progressed to a point where there are already several commercially available devices. Furthermore, a large range of next-generation devices is in development. However, current prosthetic devices are still very limited in the vision that they are able to restore. This report provides a brief overview of several recent developments in this field and discusses some of the challenges to achieveing the extensive therapeutic benefits envisioned by these new technologies.

## Engineering a visual neuroprosthesis

The concept of artificially inducing a visual sense in blind individuals is founded on our present understanding of the anatomy and physiology of the mammalian visual system and the relationship between electrical stimulation of any part of the visual pathways and the resulting visual sensations (Maynard, 2001). Hence all visual prostheses can create an artificial sense of vision by electrically activating neural cells in the visual system.

**Fig. 2:**
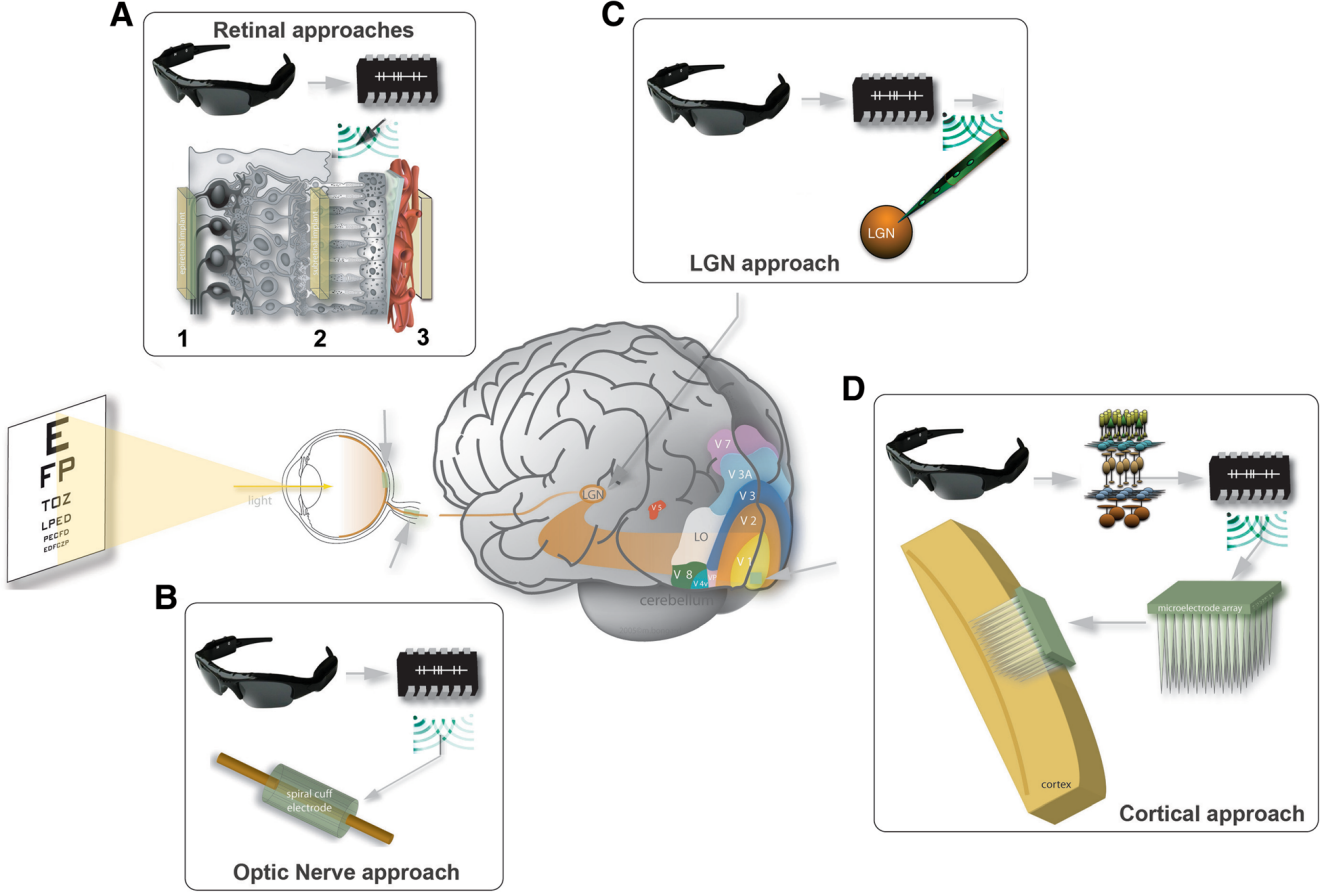
Main approaches for the design of a visual prosthesis. **a** Schematic diagram of a retina cross-section showing three methods of stimulating the output cells of the eye: 1, Epiretinal; 2, Subretinal and 3, Suprachoroidal. **b** Optic nerve based visual prosthesis. **c** Stimulation of the lateral geniculate nucleus of the thalamus (LGN). **d** Cortical approach. In general, all the approaches share a common set of components: a camera to capture images, generally mounted on more or less standard glasses; a second stage that transform the visual scene into patterns of electrical stimulation and transmits this information through a radio-frequency link to the implanted device, and an electrode array implanted at some level in the visual pathways which has to be located near the target neurons

Figure 2 shows the main current approaches for the development of visual prostheses. As blindness results from an interruption in the normal flow of signals along the visual pathways, a visual prosthesis simply has to excite the neurons of the pathway at some point beyond the damage site (Normann et al., 1996; Fernandez et al., 2005). The only requirement is that the microelectronic device should make functional contact with still functioning neural elements. Accordingly, there are now numerous corporate and academic groups around the world actively developing implants optimized for several visual pathologies, which are designed to interact specifically with the: retina (Walter et al., 1999; Zrenner, 2002; Eckmiller, 1997; Humayun et al., 2003; Rizzo 3rd et al., 2003), optic nerve (Delbeke et al., 2001; Veraart et al., 2003; Wu et al., 2010), lateral geniculate nucleus (LGN) (Pezaris & Reid, 2007; Pezaris & Eskandar, 2009; Constantinou et al., 2013; Vurro et al., 2014; Killian et al., 2016) and visual cortex (Fernandez et al., 2005; Troyk et al., 2003; Najarpour Foroushani et al., 2018; Normann & Fernandez, 2016; Fernandez et al., 2014; Normann et al., 2009; Foroushani et al., 2018).

Regardless of the differences in the approaches, most visual prostheses share a common set of components. One or more cameras provide information of the visual space located in front of a blind individual. Then this information is processed by a dedicated microelectronic device and transformed into patterns of electrical stimulation that are sent to multiple microelectrodes that can be situated at any part of the visual pathway. Therefore, most of the present systems consist of two parts, one situated outside the body (which contains the processing unit, the power supply and transmitter) and other that contains the microelectrodes and can be implanted (depending of the approach) into the eye, optic nerve or any other part of the visual pathway.

## Clinical visual prostheses

Retinal prostheses have been the most successful approach to date and several visual prostheses systems have been already approved to treat some eye diseases. In March 2011 the European Union approved Second Sight Medical Products Inc’s Argus II Retinal Prosthesis System for the treatment of patients with retinitis pigmentosa (RP), a major cause of hereditary blindness, and no functional vision. Later, in February 2013, this retinal implant received the approval of the US Food and Drug Administration for RP patients under a humanitarian device exemption. Nowadays numerous retinal prostheses have been tested in clinical trials, and three other systems have regulatory approval for patients with retinal degenerations: Alpha IMS/AMS from Retina Implant AG (Stingl et al., 2015; Stingl et al., 2012; Stingl et al., 2017), and IRIS II and PRIMA from Pixium Vision Inc. (Djilas et al., 2011; Lorach et al., 2012; Lorach et al., 2015; Butterwick et al., 2009; et al., 2015).

The trial results to date are very encouraging. Safety has been observed with all these visual prosthesis devices, and subjects wearing these systems are able to perceive light when the devices are activated, which allows them to perform some simple visual and motion tasks after a short period of training.

## Challenges and future perspectives

We are surrounded by a rich and sophisticated color environment and can hardly imagine a visual world presented in low contrast and without meaningful color (Dagnelie, 2018). However, at present, visual neuroprostheses only allow for perception of spots of light and high-contrast edges, and do not offer high enough resolution or acuity for a patient to regain a fully functional sense of vision. For example, the best visual grating acuities in ARGUS II and Alpha IMS clinical trials were 20/1260 and 20/546 respectively (Stingl et al., 2015; da Cruz et al., 2016), which is far away from the visual acuity which is required to recognize shapes, objects and letters (this topic has been reviewed by (Vurro et al., 2014; Dagnelie, 2018; Zhao et al., 2017)). What remains to be conclusively demonstrated is whether or not the visual percepts produced by these microelectronic devices can create meaningful perceptions that can be translated into functional gains such as the recognition, localization and grasping of objects, or skillful navigation in unfamiliar environments. In this framework, there are still many open technological and biological challenges that need to be resolved.

### Electrode-to-tissue Interface issues

Some key questions in this field, which are slowing down the development of more suitable devices, are also shared with other neural interfaces developed for applications such as communication and control in paralyzed patients or stimulation of auditory pathway for restoration of hearing (Normann & Fernandez, 2016). Thus, to be able to stimulate individual neurons, the microelectrodes should be located very close to the targeted cells and have dimensions similar to the neurons they are trying to stimulate. This small size requires optimized geometries and specific electrical properties to achieve a sufficient recording ability and a high relative charge transfer capacity. Thereby, if the electrodes are too large, can interact with hundreds and thousands of neurons, but if they are too small, the impedances are too high and may miss nearby neurons entirely. On the other hand, the electrodes cannot be arbitrary small, since the amount of safe charge is reduced with electrode area (Pancrazio et al., 2017; Cogan, n.d.). In addition, the substrates that host the connecting pathways to individual electrodes have to remain perfectly functional within the biological microenvironment and must be completely insulated to prevent cross-talking between electrodes (Heiduschka & Thanos, 1998; Marin & Fernandez, 2010). All of these issues impose unique constrains on the architecture, surgical techniques, and materials used in the implementation of any visual prostheses.

Other important aspects are related with the long-term viability of stimulating electrodes, which suffer electrolytic corrosion and lead to glial scarring (Fernandez et al., 2014; Marin & Fernandez, 2010; Fernandez & Botella, 2017). Thus, all biomaterials, even those thought to be highly biocompatible, provoke a biological response and some degree of encapsulation. This means that the body can tolerate them, although they are not fully compatible. Therefore, a significant goal is to improve the knowledge and understanding of the interactions between the implanted devices and the local cellular environment in order to improve the long-term biotolerability, applicability and functional perspectives of these devices. Furthermore, although hermetic packaging has been developed to meet the standards for regulatory approval, we still need new materials and improved processes to achieve robust isolation barriers able to protect the electronics during long periods of time (Jiang & Zhou, 2009; Vanhoestenberghe & Donaldson, 2013).

Furthermore, we should take into account the potential drawbacks and side effects of electrical stimulation. It is well known that information transmission in neurons lies in action potentials, which are generated by the opening of voltage-gated channels on the cellular membrane. Transient electrical stimulation can activate these channels and generate action potentials. However, this ubiquitous method of stimulation primarily works by modulation of action potential firing and, moreover, it is limited by the difficulties to confine electrical fields to target individual neurons, specific cell types or structures (Freeman et al., 2010; CC & Grill, 2002). In addition, power dissipation can become a problem, especially for large arrays of electrodes (Lazzi, 2005; Gosalia et al., 2004). To avoid some of these issues some researchers are starting to think that we need to reconsider our methods of stimulation.

Optogenetic tools (Nagel et al., 2003) have the potential to bypass many of the limitations of electrical stimulation (Delbeke et al., 2017). This technique has been validated in blind animal models of Retinitis Pigmentosa (Sahel & Roska, 2013) and is moving towards human trials for retinal prosthesis (Sahel et al., 2015; Tung et al., 2016; Sengupta et al., 2016; Barrett et al., 2016). The power of optogenetics is that specific neural sub-circuits can be targeted using gene therapy to express the light-sensitive ion channels, and high fidelity control of neural firing can be achieved. Moreover, we should also consider the possibilities offered by the optopharmacological approach. This technique is based on a new class of light-regulated drugs that are able to photo-control the activity of different ion channels and can be regarded as nanoprosthetic devices to remotely drive the endogenous receptors that remain in the cells (Izquierdo-Serra et al., 2016). Additionally, other researchers are starting to use magnetic stimulation from implantable micro-coils as an alternative to conventional micro-electrodes. Coils are attractive because they overcome many of the limitations of conventional electrodes since magnetic stimulation does not require the injection of electrical currents and asymmetric fields from coils can be used to improve focal activation of specific subsets of neurons (Lee et al., 2016).

### Delivering of information to implants

If we compare the current status of visual prostheses with that of cochlear implants (hearing restoration prostheses), there are some interesting observations. Thus, much of the recent successes of cochlear implants has been due to the advancement of signal processing techniques developed over the years and to the development of state-of-the-art multi-channel implants (Boulet et al., 2016; Jain & Vipin Ghosh, 2018; Clark, 2015). However, present visual prostheses only emulate the phototransducer aspect of the retina and lack most of the processing functionality that is found in the visual system. In this framework some researchers think that the stimulation should be performed by incorporating the neural code, that is the code that the retina uses to communicate with the brain (Nirenberg & Pandarinath, 2012). The rationale is that if we were able to convert any pattern of light falling on the retina into physiological patterns of electrical pulses it could be easier for the patients to learn and interpret the implant outputs. Preliminary experiments in mouse suggest that the incorporation of these neural coding schemes has a significant impact to improve prosthetic capabilities, well beyond what can be achieved just by increasing resolution (Nirenberg & Pandarinath, 2012), however this has yet to be proved in human clinical trials. Additionally most visual prostheses have a very limited number of electrodes that is far away of the number of cells in the retina, LGN and visual cortex. Thereby, the human retina contains approximately 120 million rods and 6 million cones that converge to about 1.2 million ganglion cells, which send their signals to the brain (Osterberg, 1935). Therefore a higher number of electrodes, able to stimulate a large number of cells, together with different levels of pre- processing depending on the place of implantation are still needed to restore a useful vision. However, this also poses several problems related mainly with data transmission, since this is one of the key factors limiting the number of electrodes. Thus, if the number of stimulating electrodes is increased, there are often considerable interference and cross-talk problems (Matteucci et al., 2016; Flores et al., 2016; Khalili Moghaddam et al., 2014).

On the other hand, we should remember that the problem is not to record a picture with a high resolution, but to send useful information to the appropriate site(s). Thus, our entire experience of the external visual world derives from the concerted activity of a restricted number of retinal ganglion cells, which have to send their information, via the optic nerve, to higher visual centers (Kolb et al., 2005). The representation has to be unequivocal and fast, in order to ensure object recognition for any single stimulus presentation within a few hundreds of milliseconds (Bialek et al., 1991; de Ruyter van Steveninck et al., 1997; Smirnakis et al., 1997). Therefore, for the success of any visual prosthesis we still need a better understanding on how the information about the external world is compressed in the retina, and how this compressed representation is encoded in spike trains that are send to the brain.

In the meantime, we should not forget that eye movements are essential for visual information processing and also affects visual acuity (Edwards et al., 2017; Yao et al., 2018; Sanefuji et al., 2018; Martinez-Conde et al., 2013). Thus, when a still image is stabilized on the retina, quickly fades and disappears from the conscious percept. To avoid it, eyes are always in constant motion, stabilizing retinal images against displacements caused by movements of the head, and taking the fovea from place to place to find interesting things to look at (Kowler, 2011). For example, when we look at a face, our gaze fixes a certain feature like the eyes, and then jumps to another feature like the mouth. Some of these movements are involuntary and appear even when we fix our gaze, which results in remapping the neural representation of a target object as well as its attentional modulation (Yao et al., 2018; Sanefuji et al., 2018). In this context a recent report shows the usefulness of eye movements for perceptual learning in a non-human primate model of artificial vision, and that higher numbers of saccades and micro-saccadic movements in a gaze-contingent prosthesis enhance performance (Killian et al., 2016). Consequently, the incorporation of saccadic and micro-saccadic movements could be useful to facilitate the learning of the patients as well as to enhance the functionality of some visual prosthetic devices. In this framework some authors are starting to incorporate these involuntary eye movements into their retinal models using different strategies: micro-saccades, drifts and tremor. The results show that transforming an originally stationary image into one that varies spatiotemporally (thus mimicking real fixational eye movements) improve feature estimation (Martínez-Álvarez et al., 2013; Olmedo-Paya et al., 2015).

Likewise, the requirements for a helpful visual prosthesis should follow from the needs and desires of blind individuals who will benefit from these devices. In this framework the visual functions that seem most important to the blind are mobility in open environments, face recognition and reading (Luo et al., 2016; Humayun et al., 2016). Consequently, the technology should be optimized and adapted to satisfy these particular needs and at the same time, take into account the opportunities of prosthetic vision for activities of daily living. Moreover, future advanced systems should also allow the customization of the functions of the visual prosthetic devices to users’ needs and capabilities.

### Eye pathologies and neural plasticity

Retinal devices are only able to restore some sight loss due to photoreceptor degeneration and are not viable for all causes of blindness. Thus, if the communication link between the eye and the brain is destroyed, as is the case for many blind patients worldwide (e.g., patients with Glaucoma or optic nerve atrophy), the device has to bypass the damaged areas. Consequently, there are compelling reasons to pursue the development of other visual prosthetic devices located at the thalamus or visual cortex, capable of restoring some useful vision in profoundly blind patients with pathologies affecting the entire retina or the optic nerve.

On the other hand, we should be aware that the mature visual system is capable of extensive reorganization as the roles of inputs and pathways are altered by visual experience and sensory loss (this topic has been recently reviewed by (Legge & STL, 2016). Thus, the visual cortex retains the capacity for experience-dependent changes throughout life and as a consequence each cortical area can alter its function in accordance to immediate perceptual demands (Legge & STL, 2016; Gilbert et al., 2009; Karni & Bertini, 1997). In this context, several studies have suggested that in some patients the occipital parts of the brain that sighted subjects utilize to process visual information are transformed and can be utilized to process tactile and auditory stimuli (Fernandez et al., 2005; Bavelier & Neville, 2002; Merabet et al., 2005; Ptito & Kupers, 2005; Bernabeu et al., 2009; Beyeler et al., 2017). Thereby there is considerable evidence showing diverse adaptive and compensatory changes that occur within the brain following the loss of sight. These changes are the inevitable consequence of sensory deprivation and allow blind subjects to extract greater information from touch and hearing. However, it is important to note that not all these neuroplastic changes are necessarily beneficial, since they can also limit the degree of adaptation, or even be maladaptive (Merabet et al., 2007). Consequently, the modulation of the neuroplasticity and a better understanding of these adaptive changes are crucial for the success of any visual neuroprosthesis.

### Patient selection and visual rehabilitation

There are no strict standardized criteria for recommending, accepting or rejecting a candidate for a particular visual prosthetic device. Generally, a choice could be made between different approaches and/or rehabilitation procedures depending on availability and efficacy (Veraart et al., 2004; Dagnelie, 2006; Dowling, 2005), nevertheless clear indications as well as pre-surgical protocols and improved methods for predicting success need to be developed (Fernandez et al., 2005; Merabet et al., 2007; Dagnelie, 2008). This issue is further complicated because at present it is not possible to predict success with a given visual implant in a specific person. Clearly, our knowledge regarding visual system anatomy and function may allow for crude bio-inspired models and strategies of stimulation. However, what has not been discussed is how the type, onset, duration and temporal profile of an individual’s visual loss may have repercussions on the success of a particular visual prosthesis device. On the other hand to decide at what point in a subject’s history of sight loss is most suitable is also a difficult double-faced ethical decision (Merabet et al., 2007; Veraart et al., 2004).

Finally, there is also a considerable gap between visual prosthesis device implementation and the rehabilitative challenges that arise from these new technologies. Thus, although prosthetic vision rehabilitation has many parallels with low-vision rehabilitation, also differs in many ways from native vision (Jeter et al., 2017). As a consequence, the modest success achieved to date with human experimentation is not merely limited by the technical issues that remain to be solved but is also significantly related to our limited knowledge on how to communicate with a visually deprived brain. Thus, the rehabilitation of the blind using artificial devices is a very complex issue, requiring intimate collaborations among basic scientists, engineers, health care professionals, educators and rehabilitative experts towards a more effective use of their restored vision.

## Conclusions

The possibility of restoring vision to the blind is closer than ever. Retinal implants have already showed some promising results, and in the next years we will likely see significant strides in progress in other visual prosthetic systems as technological, surgical and rehabilitative techniques all improve. However, there are still a number of open relevant considerations. We hope that the progresses in medical technologies, neuroscience, electronics, material science and bioengineering, together with the increase of intelligence in these visual neuroprosthetic devices and the involvement of experts on vision rehabilitation, will foster the development of new and improved custom-tailored neuroelectronic systems for restoring a functional sight in many blind persons.

## Author information

Eduardo Fernandez received a M.D. degree at the University of Alicante (1986) and a Ph.D. in Neuroscience (1990). He is currently Full Professor and Chairman of the Department of Histology and Anatomy at the University Miguel Hernandez de Elche (Spain), Director of the Neural Engineering Division of CIBER-BBN Networking Centre on Bioengineering, Biomaterials and Nanomedicine (Spain), and Adjunct Professor at John Moran Eye Center (University of Utah, USA). In the latest years he has been coordinating several projects to demonstrate the feasibility of a neuroprosthesis interfaced with the occipital cortex, as a means through which a limited but useful sense of vision could be restored to profoundly blind people.
